# Care and Feeding of the Endocannabinoid System: A Systematic Review of Potential Clinical Interventions that Upregulate the Endocannabinoid System

**DOI:** 10.1371/journal.pone.0089566

**Published:** 2014-03-12

**Authors:** John M. McPartland, Geoffrey W. Guy, Vincenzo Di Marzo

**Affiliations:** 1 GW Pharmaceuticals, Porton Down Science Park, Salisbury, Wiltshire, United Kingdom; 2 Department of Family Medicine, University of Vermont, Burlington, Vermont, United States of America; 3 Endocannabinoid Research Group, Istituto di Chimica Biomoleculare, CNR, Via Campi Flegrei, Pozzuoli, Napoli, Italy; St. Joseph's Hospital and Medical Center, United States of America

## Abstract

**Background:**

The “classic” endocannabinoid (eCB) system includes the cannabinoid receptors CB_1_ and CB_2_, the eCB ligands anandamide (AEA) and 2-arachidonoylglycerol (2-AG), and their metabolic enzymes. An emerging literature documents the “eCB deficiency syndrome” as an etiology in migraine, fibromyalgia, irritable bowel syndrome, psychological disorders, and other conditions. We performed a systematic review of clinical interventions that enhance the eCB system—ways to upregulate cannabinoid receptors, increase ligand synthesis, or inhibit ligand degradation.

**Methodology/Principal Findings:**

We searched PubMed for clinical trials, observational studies, and preclinical research. Data synthesis was qualitative. Exclusion criteria limited the results to 184 *in vitro* studies, 102 *in vivo* animal studies, and 36 human studies. Evidence indicates that several classes of pharmaceuticals upregulate the eCB system, including analgesics (acetaminophen, non-steroidal anti-inflammatory drugs, opioids, glucocorticoids), antidepressants, antipsychotics, anxiolytics, and anticonvulsants. Clinical interventions characterized as “complementary and alternative medicine” also upregulate the eCB system: massage and manipulation, acupuncture, dietary supplements, and herbal medicines. Lifestyle modification (diet, weight control, exercise, and the use of psychoactive substances—alcohol, tobacco, coffee, cannabis) also modulate the eCB system.

**Conclusions/Significance:**

Few clinical trials have assessed interventions that upregulate the eCB system. Many preclinical studies point to other potential approaches; human trials are needed to explore these promising interventions.

## Introduction

The endocannabinoid (eCB) system consists of receptors, endogenous ligands, and ligand metabolic enzymes. Metaphorically the eCB system represents a microcosm of psychoneuroimmunology or mind-body medicine. Cannabinoid receptor 1 (CB_1_) is the most abundant G protein-coupled receptor expressed in the brain, with particularly dense expression in (rank order): the substantia nigra, globus pallidus, hippocampus, cerebral cortex, putamen, caudate, cerebellum, and amygdala [1]. CB_1_ is also expressed in non-neuronal cells, such as adipocytes and hepatocytes, and in musculoskeletal tissues. Cannabinoid receptor 2 (CB_2_) is principally associated with cells governing immune function, although it may also be expressed in the central nervous [2], [3].

The quintessential eCB ligands are *N*-arachidonylethanolamide (anandamide, AEA) and *sn*-2-arachidonoylglycerol (2-AG). AEA and 2-AG are released upon demand from cell membrane-embedded phospholipid precursors. The primary biosynthetic enzyme of AEA is *N*-acyl-phosphatidylethanolamine phospholipase D (NAPE-PLD). 2-AG is biosynthesized by two isoforms of diacylglycerol lipase, DAGLα and DAGLβ. AEA and 2-AG work in a homeostatic fashion, thus they are broken down after they activate CB_1_ or CB_2_. AEA is catabolized primarily by fatty acid amide hydrolase 1 (FAAH1), and 2-AG is catabolized by monoacylglycerol lipase (MAGL), and, to a lesser extent, α,β-hydrolase-6 (ABHD-6), cyclooxygenase 2 (COX2), and FAAH1.

This “classic eCB system” has expanded with the discovery of secondary receptors, ligands, and ligand metabolic enzymes [4]. For example, AEA, 2-AG, *N*-arachidonoyl glycine (NAGly) and the phytocannabinoids Δ^9^-tetrahydrocannabinol (THC) and cannabidiol (CBD) may also serve, to different extents, as ligands at GPR55, GPR18, GPR119, and several transient receptor potential ion channels (*e.g.*, TRPV1, TRPV2, TRPA1, TRPM8). The effects of AEA and 2-AG can be enhanced by “entourage compounds” that inhibit their hydrolysis via substrate competition, and thereby prolong their action. Entourage compounds include *N*-palmitylethanolamide (PEA), *N*-oleoylethanolamide (SEA), and *cis*-9-octadecenoamide (OEA, oleamide).

The eCB system's salient homeostatic roles have been summarized as, “relax, eat, sleep, forget, and protect” [5]. It modulates embryological development, neural plasticity, neuroprotection, immunity and inflammation, apoptosis and carcinogenesis, pain and emotional memory, and most importantly from the viewpoint of recent drug development: hunger, feeding, and metabolism. Obese individuals seem to display an increased eCB tone, driving CB_1_ activation in a chronic, feed-forward dysfunction (reviewed by [6]). An antagonist or inverse agonist of CB_1_ called rimonabant (*aka*, SR141716 in preclinical studies) was approved for the treatment of obesity. It was subsequently withdrawn from the market due to adverse effects [7].

Other diseases are associated with suboptimal functioning of the eCB system. Russo [8] proposed that migraine, fibromyalgia, irritable bowel syndrome, and related conditions represent CEDS, “clinical endocannabinoid deficiency syndromes.” Fride [9] speculated that a dysfunctional eCB system in infants contributes to “failure to thrive” syndrome. Hill and Gorzalka [10] hypothesized that deficient eCB signaling could be involved in the pathogenesis of depressive illnesses. In human studies, eCB system deficiencies have been implicated in uncompensated schizophrenia [11], migraine [12], multiple sclerosis [13], Huntington's [14], [15], uncompensated Parkinson's [16], irritable bowel syndrome [17], uncompensated anorexia [18], and chronic motion sickness [19].

Correcting CEDS may be accomplished via at least three molecular mechanisms: 1. augmenting eCB ligand biosynthesis; 2. decreasing eCB ligand degradation; 3. augmenting or decreasing receptor density or function. Clinical interventions for CEDS are largely unknown; this provided a rationale for reviewing potential clinical approaches. The paucity of human clinical trials led us to include preclinical studies in a systematic review. A systematic review uses an objective, transparent approach for research synthesis, with the aim of minimizing bias. Systematic reviews usually analyze human clinical trials, but the methodology can be applied to preclinical studies [20], [21]. We previously conducted a systematic review of *in vitro* CB_1_ ligand binding affinity and receptor distribution [22]. The review has alerted others to inter-species differences in preclinical studies, and other methodological issues (*e.g.*, [23]).

Potential clinical interventions (intervention groups) include pharmaceutical drugs, such as analgesics (acetaminophen, nonsteroidal anti-inflammatory drugs, opiates, glucocorticoids), antidepressants, antipsychotics, anxiolytic agents, and anticonvulsants. We also investigated therapeutic approaches classified as “complementary and alternative medicine” (CAM). The National Center for Complementary and Alternative Medicine (NCCAM) defines CAM as “a group of diverse medical and healthcare systems, practices, and products, that are not currently part of conventional medicine” (http://nccam.nih.gov/health/whatiscam/). The NCCAM categorizes CAM practices into three broad groups: “natural products” (dietary supplements and herbal remedies), “mind and body medicine” (meditation, yoga, and acupuncture), and “body-based practices” (massage, spinal manipulation). For the purposes of this review, we add “lifestyle modifications,” including diet, weight control, exercise, and commonly-used psychoactive substances—alcohol, tobacco, coffee, and cannabis.

## Methods

### Data Sources and Search Parameters

This review followed the guidelines proposed by PRISMA, the Preferred Reporting Items for Systematic Reviews and Meta-Analyses [24], see Checklist S1. PubMed (www.ncbi.nlm.nih.gov/pubmed/) was searched through March 2013 using three MeSH keywords: endocannabinoids, cannabinoids, cannabinoid receptors. Each keyword was entered in a boolean combination with each of the intervention groups listed in the previous paragraph. Titles and abstracts of identified articles in all languages were screened for inclusion and exclusion criteria. We included randomized clinical trials, observational studies, and preclinical research on model organisms and *in vitro* studies. We excluded redundant articles that used identical methods and reported parallel results, or review articles that presented duplicate information.

Because this review focuses upon clinical interventions affecting the eCB system, we deemed as irrelevant (and excluded) articles that described the reverse scenario, such as eCB ligands modulating opioid receptors, THC enhancing tobacco or alcohol abuse, etc. Retrieved articles were scanned for supporting citations, and antecedent sources were retrieved and screened for inclusion and exclusion criteria. In addition, we checked reference lists of relevant narrative reviews.

### Data Selection, Abstraction, and Synthesis

All three authors selected studies for inclusion and exclusion; the first author abstracted all data, the second and third authors arbitrated uncertainties and disagreements. We undertook a qualitative synthesis across studies because there was substantial heterogeneity with respect to research methodologies amongst the identified articles—ranging from randomized clinical trials, observational studies, and preclinical research on model organisms and *in vitro* studies. The substantial heterogeneity amongst these methodologies precluded a single metric of quality assessment.

Many studies utilized *in vitro* measures of receptor density and signal transduction, as differences in means before- and after-interventions. Briefly: assays for CB_1_/CB_2_ receptor density include autoradiography with tritiated ligands (usually [^3^H]CP55,940 or [^3^H]SR141716), Western blot or immunostaining with antibodies to CB_1_/CB_2_ proteins, and Northern blot with radio-labeled or fluorescent riboprobes for CB_1_/CB_2_ mRNA. Signal transduction studies measure cannabinoid-stimulated inhibition of adenylyl cyclase, cannabinoid-stimulated [^35^S]GTP*γ*S binding, or electrophysiological assays of *ex vivo* brain slices. Electrophysiological studies include depolarization-induced suppression of excitation (DSE, via glutamatergic synapses), depolarization-induced suppression of inhibition (DSI, via GABAergic synapses), long-term depression of excitatory synaptic transmission (LTDE, via glutamatergic synapses), or long-term depression of inhibitory synaptic transmission (LTDI, via GABAergic synapsis).

Publication bias was addressed by asking investigators to contribute unpublished studies. Clinical interventions (intervention groups) with five or more studies are provided with an interpretive summary at the end of the section (*e.g.*, the sections on NSAIDs, glucocorticoids, opiates, etc.).

## Results and Discussion

**Figure 1:**
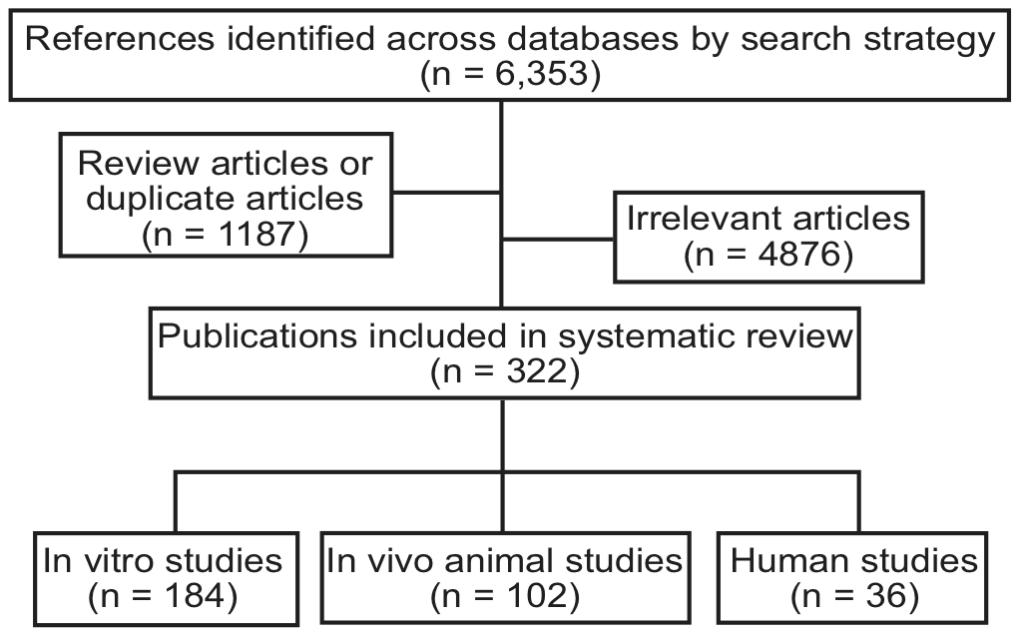
Selection process for study inclusion.

The search algorithm identified 6,353 potentially relevant articles. The majority of these were irrelevant. For example, combining the three MeSH keywords with “alcohol” generated 2450 hits, many of which concerned the relationship between alcohol and cannabis in motor vehicle accidents or suicides. Only 322 articles met the predefined selection criteria for relevance. See Figure 1 for a flowchart of articles included in this review. Few randomized clinical trials have been conducted on our topic; most of the articles concerned preclinical research. Fewer studies measured the effects of clinical interventions on CB_1_ expression in humans. This is because the measurement of CB_1_ expression requires positron emission tomography (PET) or brain biopsies. Although cannabinoid radioligands for PET scans are available, few PET studies on clinical interventions have been completed. Ethical issues circumscribe brain biopsies in living humans. A few studies measured postmortem CB_1_ expression.

The use of PubMed as a stand-alone search engine may have generated bias regarding CAM practices. McPartand and Pruitt [25] used PubMed to compile a review of clinical trials regarding the CAM herbal medicine *Serenoa repens*; PubMed yielded only 33% of articles that they subsequently obtained by screening retrieved articles for supporting citations. Expanding our search by screening retrieved articles for supporting citations improved the yield, as it did in the *Serenoa* review.

The quality of *in vitro* studies such as [^3^H]CP55,940 binding at CB_1_ was generally high, for example, PMSF was used when appropriate. Methods used in two electrophysiology studies were controversial, and the studies were removed after urging by our manuscript reviewers. The quality of some rodent models of behavior was also questionable. Rather than judge their translational validity—a contentious issue [26]—we have named the specific behavioral assays in each study, allowing the reader to pass judgment.

### Pharmaceutical drugs

#### Non-steroidal anti-inflammatory agents (NSAIDs)

**Figure 2:**
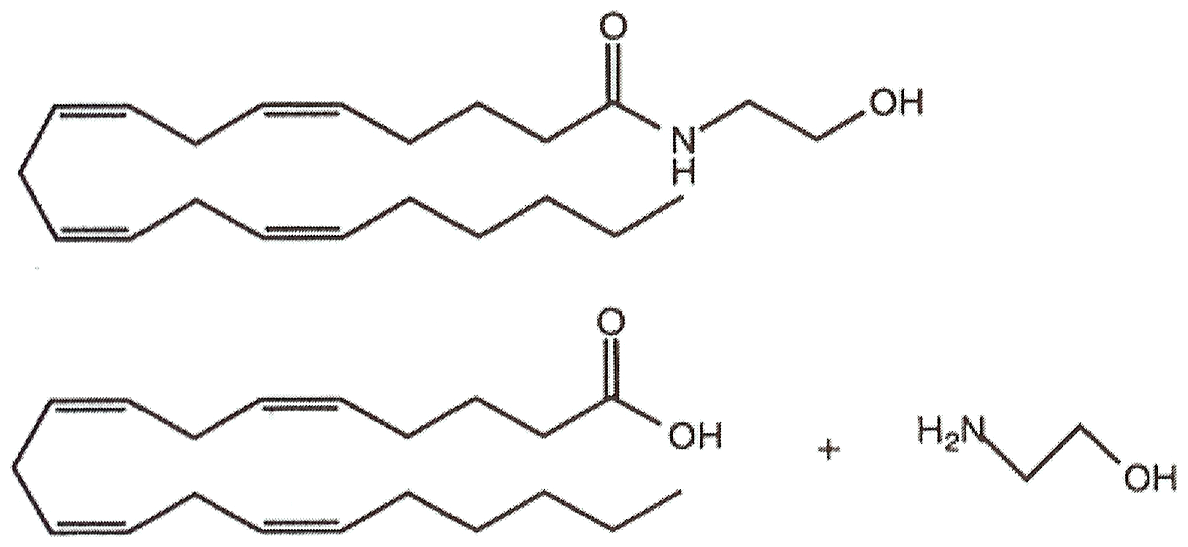
Anandamide (top figure) is metabolized into arachidonic acid and ethanolamine (bottom figures).

NSAIDs inhibit two cyclooxygenase (COX) enzymes, COX1 and COX2, and thereby block the conversion of arachidonic acid (AA) into inflammatory prostaglandins. Ibuprofen, ketorolac, and flurbiprofen also block the hydrolysis of AEA into arachidonic acid and ethanolamine [27]. See Figure 2. A binding site for some NSAIDs on FAAH has also been identified [28]. NSAID inhibition of COX2 blocks the metabolism of AEA and 2-AG into prostaglandin ethanolamides (PG-EAs) and prostaglandin glycerol esters (PG-GEs), respectively [29]. PG-EAs and PG-GEs *increase* the frequency of miniature inhibitory postsynaptic currents (mIPSCs) in primary cultured mouse hippocampal neurons, an effect opposite to that of their parent molecules [30].

Prostaglandin E_2_ glycerol ester (PGE_2_-GE), a COX2 metabolite of 2-AG, induced mechanical allodynia and thermal hyperalgesia in rat paws [31]. PGF_2_α-EA, a COX2 metabolite of AEA, was found in the spinal cord of mice with carrageenan-induced knee inflammation. PGF_2_α-EA contributed to pain perception and dorsal horn nociceptive neuron hyperactivity, thus providing a rationale for the combined use of COX2 and FAAH1 inhibitors against inflammatory pain [32].

Electrophysiology studies of rat hippocampal cells showed that meloxicam and nimesulide prolonged and increased DSI; that is to say, the COX2 inhibitors potentiated synaptic 2-AG release and CB_1_ signaling [33]. Consistent with this, intrathecally applied indomethacin enhanced eCB-mediated antinociception in mice that was blocked by the CB_1_ antagonist AM251 [34]. Intrathecally applied flurbiprofen produced a similar eCB-dependent antinociception in the rat formalin test [35].

Combining NSAIDs with cannabinoids (either eCBs or exogenous cannabinoids) produces additive or synergistic effects. A sub-effective dose of WIN55,212-2 became fully antinociceptive following administration of indomethacin in rats [36]. A local injection of ibuprofen plus AEA in the rat formalin test produced synergistic antinociceptive effects involving both CB_1_ and CB_2_ [37]. The FAAH inhibitor URB937, when coadministered to mice with indomethacin, produced a synergistic reduction in pain-related behaviors [38]. Furthermore, URB937 reduced the number and severity of gastric lesions produced by indomethacin. One contrary study showed that THC's decrease in intraocular pressure was partially blocked by indomethacin in rabbits [39].

In a small human study, the administration of indomethacin antagonized marijuana effects [40]. Yet a high dose of ibuprofen may cause sedation, possibly a cannabimimetic effect [41]. Clinical anecdotes of NSAIDs eliciting cannabimimetic effects have been reported; the individuals are usually familiar with the effects of cannabis, and usually females [42].

In summary, preclinical studies indicate that some NSAIDs inhibit FAAH and enhance the activity of eCBs, phytocannabinoids, and synthetic cannabinoids. Combinational effects may be particularly relevant at peripheral sites, such as the peripheral terminals of nociceptors.

#### Acetaminophen

Acetaminophen (paracetamol), following deacetylation to its metabolite *p*-aminophenol, is conjugated with AA to form *N*-arachidonoylphenolamine (NAP, *aka* AM404). It is likely that deacetylation takes place mainly in the liver, and conjugation occurs in the central nervous system. NAP blocks the breakdown of AEA by FAAH, inhibits COX1 and COX2, and acts as a TRPV1 agonist [43]. The analgesic activity of acetaminophen in rats is blocked by CB_1_ or CB_2_ antagonists [44], [45]. Analgesic activity is also lost in CB_1_^−/−^ knockout mice [46]. A sub-effective dose of the synthetic cannabinoid WIN55,212-2 became effective following intracisternal administration of acetaminophen in rats [36]. A sub-effective dose of AEA in mice became anxiolytic in the Vogel conflict test and the social interaction test when co-administered with acetaminophen; the effect was blocked by the CB_1_ antagonist AM251 [47].

Small amounts of acetaminophen are also metabolized via the cytochrome P-450 pathway into *N*-acetyl-*p*-benzoquinone imine (NAPQI). Intrathecal administration of NAPQI activates TRPA1 and imparts antinociception in the mouse hot-plate test, and a similar action is found for Δ^9^-tetrahydrocannabiorcol. These effects are lost in Trpa1(−/−) mice [48].

In summary, preclinical studies indicate that acetaminophen enhances the activity of eCBs and synthetic cannabinoids in rodents. Why acetaminophen fails to elicit cannabimimetic effects in humans is unknown. Acetaminophen-cannabinoid drug interactions may be species-specific; Gould *et al.* [49] demonstrated strain-specific differences in mice. They suggested that other indirect actions of acetaminophen, including 5-HT receptor agonism, may outweigh any CB_1_ mediated effects in some mouse strains.

#### Glucocorticoids

The distribution of glucocorticoid receptors (GRs) and CB_1_ overlap substantially in the central nervous system and other tissues, as do GRs and CB_2_ in immune cells. Dual activation of GRs and CBs may participate in glucocorticoid-mediated anti-inflammatory activity, immune suppression, insulin resistance, and acute psychoactive effects. In a rat model of spinal nerve injury (sciatic nerve constriction with suture loops), the GR receptor agonist dexamethasone increased CB_1_ density after spinal nerve injury, which suggests that CB_1_ is a downstream target for GR actions [50]. Glucocorticoid administration also induced CB_1_ expression in bone in mice [51] and rats [52].

The *acute* administration of glucocorticoids may shift AA metabolism toward eCB synthesis in parts of the brain. Electrophysiological studies of rat hypothalamic slices demonstrated that adding dexamethasone or corticosterone to slice baths caused a rapid suppression of synaptic activity, characterized as glucocorticoid-induced, eCB-mediated suppression of synaptic excitation (GSE). GSE was blocked by CB_1_ antagonists, indicating that eCB release mediated GSE [53]. A follow-up study demonstrated that GSE correlated with increased levels of AEA and 2-AG [54]. The same group found no changes in AEA and 2-AG after exposure of cerebellar slices to dexamethasone. In hypothalamic slices, GSE could be blocked by leptin, suggesting that GSE is a nutritional state-sensitive mechanism [55]. Dexamethasone enhanced eCB-mediated GSE by inhibiting COX2 in dorsal raphe serotonin neurons [56].

Corticosterone administration increased AEA levels in several rat limbic structures (amygdala, hippocampus, hypothalamus), but not the prefrontal cortex. 2-AG levels were only elevated in the hypothalamus [57]. The same group conducted an *ex vivo* study of the rat medial prefrontal cortex (mPFC). Bath application of corticosterone to mPFC slices suppressed GABA release onto principal neurons in the prelimbic region, which was prevented by application of the CB_1_ antagonist AM251 [58]. This indicates local recruitment of eCB signaling, probably through 2-AG. A previous study of rats receiving a single dose of corticosterone detected no change in 2-AG and a reduction of AEA in hippocampal homogenates [59]. Corticosterone increased hippocampal levels of 2-AG in rats; the impairment of contextual fear memory by corticosterone was blocked by the CB_1_ antagonist AM251 [60].

*Chronic* exposure to glucocorticoids downregulates the eCB system. Chronic corticosterone administration decreased CB_1_ densities in rat hippocampus [59] and mouse hippocampus and amygdala [61]. Chronic corticosterone administration in male rats led to visceral hyperalgesia in response to colorectal distension, accompanied by increased AEA, decreased CB_1_ expression, and increased TRPV1 expression in dorsal root ganglia. Co-treatment with the corticoid receptor antagonist RU-486 prevented these changes [62].

In summary, preclinical rodent studies indicate that *acute* glucocorticoid administration enhances the activity of eCBs. The clinical phenomenon of acute “corticosteroid mania” may have a cannabimimetic component. *Chronic* exposure to glucocorticoids downregulates the eCB system, a scenario consistent with chronic stress, which we review below.

#### Opiates

Naloxone, a μ-opioid receptor antagonist, inhibited THC-induced Fos immunoreactivity in several regions of the rat central nervous system, including the ventral tegmental area, hypothalamus, caudate-putamen, and periaqueductal grey. Conversely, naloxone and THC had an additive effect on Fos immunoreactivity in the amygdala, stria terminalis, insular cortex, and paraventricular nucleus of the thalamus [63].

Short-term co-administration of morphine with THC caused an upregulation of CB_1_ protein in the spinal column of rats, far greater than THC or morphine given alone [64]. A rodent study of chronic but voluntary intake of opiates (rats self-administering heroin) enhanced [^3^H]CP55,940 binding in the amygdala and ventral tegmental area, plus a marked increase in cannabinoid-stimulated [^35^S]GTP*γ*S binding in the nucleus accumbens, caudate putamen, and amygdala [65]. Superperfusion of *ex vivo* rat nucleus accumbens slices with 4-aminopyridine and NMDA released glutamate and GABA, respectively, and either morphine or the CB_1_ agonist HU210 predictably inhibited these responses. Combining HU210 and morphine caused a synergistic inhibition of GABA release, but a non-additive response in glutamate release [66].

Chronic morphine exposure in rats caused a reduction in hippocampal and cerebellar CB_1_ density measured with [^3^H]CP55,940, and a strong reduction in CP55,940-stimulated [^35^S]GTP*γ*S binding; 2-AG contents were also reduced [67]. Another rat study showed that chronic morphine exposure caused variable, regionally-specific modulations in [^3^H]CP55,940 binding and CB_1_ mRNA levels; CB_1_ upregulated in some regions and dowregulated in other regions [68]. In human CB_1_-transfected HEK293 cells, morphine induced a desensitization of the μ-opioid receptor and heterologous desensitization of CB_1_, demonstrated by a reduction in WIN55212-2-induced [Ca^2+^]_i_ release [69]. μ-opioid receptor knockout mice showed a dramatic reduction in WIN55212-2-stimulated [^35^S]GTP*γ*S binding [70]. In human SH-SY5Y neuroblastoma cells, sequential activation of CB_1_ and δ-opioid receptor produced synergistic elevations of intracellular Ca^2+^, a response that each receptor alone did not trigger in an efficacious way [71].

In behavioral studies, heroin reinstated “drug-seeking” behavior for WIN55,212-2 in rats [72]. Morphine did the same for THC in monkeys [73]. The rewarding effects of THC, measured by conditioned place-preference, were reversed by naloxone in rats [74]. In rats trained to discriminate THC, morphine administration markedly potentiated the THC discriminative stimulus [75]. Morphine or codeine potentiated THC-induced antinociception and analgesia in mice and rats [76]–[79]; inactive doses of the drugs in combination produce potent, synergistic analgesia [80]. Synergistic analgesia was confirmed in an isobolographic analysis [64]. Historically this is the first isobolographic analysis of a cannabinoid since the days Walter Siegfried Loewe, who invented the isobologram to test drug combinations for synergy [81]. Loewe demonstrated synergy generated by cannabis extracts combined with other drugs [82], [83], as well as synergy generated amongst the individual components within cannabis itself [84], [85].

Normal men subjected to a thermal pain stimulus did not experience analgesia from a low dose of nabilone (a synthetic THC analogue), or a low dose of morphine. But co-administration of the drugs produced an analgesic effect [86].

Endorphins (endogenous opioids) enhance the effects of cannabinoids: Administering a low dose of THC to rats produced an anxiolytic response in the light-dark box test, which was abolished by beta-funaltrexamine, a μ-opioid receptor antagonist [87]. In rats trained to discriminate THC, microinjection of β-endorphin into the ventral tegmental area potentiated the THC discriminative stimulus [75]. Enkephalins (endogenous opioids) also enhance the effects of THC: the inhibition of encephalin-degrading enzymes augmented THC-induced antinociception in mice, an effect blocked by either rimonabant or naloxone [88]. Naltrexone, a μ- and κ-opioid receptor antagonist, significantly increased many of the “positive” subjective effects of oral THC [89] and smoked cannabis [90] in marijuana smokers. These results suggest that endogenous opioids contribute to the effects of cannabis.

In summary, preclinical studies and clinical trials indicate that *acute* opiate administration enhances the activity of eCBs, phytocannabinoids, and synthetic cannabinoids. Acute opiates may also upregulate CB_1_ expression. *Chronic* opiate administration, however, may have a deleterious effect on the eCB system.

#### Antidepressant drugs

Serotonin selective uptake inhibitors (SSRIs), tricyclic antidepressants (TCAs) and monoamine oxidase inhibitors (MAOIs) are the most commonly prescribed antidepressant drugs. Treatment with fluoxetine, the archetypal SSRI, potentiated THC-induced hypothermia in rats [91], but did not change THC-induced behavioral effects—freezing behavior, social interaction or exploration, and preference for outer or inner zones [92]. Fluoxetine increased CB_1_ binding density in the prefrontal cortex, without altering AEA or 2-AG levels in rat brains [93]. Chronic fluoxetine also increased WIN55212-2-stimulated [^35^S]GTP*γ*S binding in the rat prefrontal cortex [94]. Conversely, treatment with citalopram reduced HU210-stimulated [^35^S]GTP*γ*S binding in the rat hypothalamus and hippocampus [95].

Treatment with fluoxetine prevented synaptic defects in mice induced by chronic unpredictable stress (the CUS protocol included inversion of day/night light cycle, 45° tilted cage, cage rotation, tube restraint, predator sounds, strobe lights, food and water deprivation, cold environment, and wet bedding), and CUS preserved eCB- and WIN55,212-2-stimulated CB_1_ signaling [96]. In the hands of Mato *et al.* [97], fluoxetine in rats enhanced the inhibition of adenylyl cyclase by WIN55212-2, but did not alter WIN55212-2-stimulated [^35^S]GTP*γ*S binding or CB_1_ density measured with [^3^H]CP55,940. They proposed that fluoxetine enhanced WIN55212-2 signaling through Gαi2 and Gαi3 subunits and not through Gαo subunits.

Treatment with the TCA desipramine increased CB_1_ binding density in the hippocampus and hypothalamus, without significantly altering AEA or 2-AG levels in rat brains [98]. The CUS protocol altered CB_1_ density in rat brains, and these changes were attenuated by concurrent treatment with imipramine [99]. Desipramine-induced weight gain was reduced by cotreatment with SR141716A, suggesting an eCB pathway [100].

Treatment with the MAOI tranylcypromine increased CB_1_ binding density in the prefrontal cortex and hippocampus, and increased 2-AG but decreased AEA levels in the prefrontal cortex [93]. Repeated electroconvulsive shock treatment (EST) for depression produced complex and regionally specific effects. Generally EST downregulated CB_1_ binding density and AEA levels in the cortex, but enhanced cannabinoid-stimulated [^35^S]GTP*γ*S binding in the amygdala [101].

In summary, the effects of antidepressant drugs or treatments upon the eCB system are not definitive, but likely result in CB_1_ upregulation, at least in some brain regions. Preclinical studies suggest agonist trafficking may be responsible for variable responses.

#### Antipsychotic drugs

First-generation antipsychotic drugs, such as haloperidol and chlorpromazine (thorazine), are dopamine D_2_ receptor inverse agonist. Second-generation “atypical” antipsychotics (*e.g.*, risperidone, olanzapine, clozapine, and aripiprazole) antagonize D_2_ and 5-HT_2A_, and also target other neuroreceptors. Acute administration of chlorpromazine enhanced the hypothermic response to THC [102]. Subchronic administration of haloperidol increased CB_1_ density in rat brains, indicated by increased binding of [^3^H]CP55,940 in the substantia nigra>globus pallidus>striatum. Subchronic haloperidol also potentiated CP55,940-stimulated [^35^S]GTP*γ*S binding in the substantia nigra [103]. Sundram *et al.* [104] confirmed haloperidol's effects on [^3^H]CP55,940 binding, and obtained similar results with chlorpromazine and olanzapine. In monkeys trained to discriminate THC, haloperidol sensitized the THC discriminative stimulus [105]. Risperidone increased [^3^H]CP55,940 binding in rat brain without altering CB_1_ mRNA levels [106]. Four weeks of aripiprazole upregulated CB_1_ in rat frontal cortex [107]. Clozapine decreased [^3^H]CP55,940 binding in rat brain [104], and attenuated THC-induced disruption of spatial working memory in the rat radial maze task [108].

Several researchers have proposed that CB_1_ upregulation during antipsychotic drug treatment may explain appetite enhancement, weight gain, and CB_1_ supersensitivity. D'Souza *et al.* [109] conducted a double-blind study on the effects of adding haloperidol to THC. Compared to THC alone, the combination of drugs significantly worsened verbal recall, distractibility, and vigilance scores. The drug combination did not affect other testing parameters, such as euphoric effects and motor outcomes. Another double-blind study showed that haloperidol reversed THC-induced increases in the Positive and Negative Syndrome Scale (used for measuring symptom severity in schizophrenia), but did not affect the THC-induced “high” in healthy male volunteers [110]. A double-blind study in healthy male volunteers showed that olanzapine reduced the effects of THC as measured on the positive and negative syndrome scale, and the visual analogue scale for psychedelic effects, but the reduction fell short of statistical significance, *p* = 0.67 [111].

In summary, antipsychotic drugs likely upregulate CB_1_ expression in parts of the rodent brain. In human clinical studies, antipsychotic drugs do not affect THC-induced “high” or “euphoria,” but dampen dysphoria and worsen verbal recall and distractibility.

#### Anxiolytics, sedatives, and anesthetics

Diazepam is used for treating anxiety, insomnia, muscle spasms, and seizure disorders. Combining diazepam with WIN55212-2 produced a supra-additive anxiolytic effect in the rat elevated plus maze test; combining diazepam with the FAAH inhibitor URB597 also led to a supra-additive effect; coadministration of diazepam with the CB_1_ antagonist AM251 attenuated diazepam's anxiolytic effect [112]. These findings might be explained by the observation that both chronic and, particularly, acute administration of diazepam to mice is accompanied by strong elevation of brain eCB levels [113].

The anxiolytic and sedative effects of alprazolam were also attenuated by AM251 in mouse behavioral assays (light-dark box test, neurological severity score, and step-down inhibitory avoidance test) [114]. Surprisingly, however, the administration of alprazolam reduced WIN55212-2-stimulated [^35^S]GTP*γ*S binding in mouse amygdala and hippocampus [114]. CB_1_^−/−^ knockout mice showed impaired anxiolytic responses to both buspirone and bromazepam in light/dark box, elevated plus maze, and social interaction tests [115]. N-arachidonoyl-serotonin (AA-5-HT), a dual FAAH/TRPV1 blocker, imparted anxiolytic effects in the mouse elevated plus maze assay [116].

A sub-effective dose of THC given to mice caused catelpsy in the horizontal bar test after sub-effective doses of either flurazepam or baclofen were added [117]. The beta-adrenergic blocking agent propranolol causes mild sedation, but pretreatment with propranolol blocked cannabis-induced cardiovascular effects and learning impairment in a small clinical trial [118].

General anesthesia (midazolam, sufentanil, isoflurane, and sufentanil) resulted in decreased serum AEA in patients stressed by the anticipation of cardiac surgery [119]. The dissociative anesthetic phencyclidine (PCP) impairs rotarod performance and open-field behavior in rats, effects shared by THC; combining the two caused supra-additive results [120]. Low-grade Mexican marijuana was adultered with PCP and marketed as “superweed” in the 1970s [121]. Nitrious oxide and THC both increase pain threshold in the tail-flick and hot-plate test, and their combination caused supra-additive effects [122].

#### Anticonvulsants

Combining diazepam with WIN55212-2 produced a supra-additive anticonvulsant effect in rats; combining diazepam with the FAAH inhibitor URB597 also led to a synergistic effect; coadministration of diazepam with the CB_1_ receptor antagonist AM251 attenuated the anticonvulsant effect of diazepam [123]. Chronic administration of valproate in rats increased CB_1_ binding of the PET scan tracer [^18^F]MK-9470; this was not seen with levetiracetam [124]. Tiagabine, an anticonvulsant GABA reuptake inhibitor, augmented THC-induced catalepsy [117] but not antinociception or hypothermia [125]. In a human study, tiagabine augmented THC discrimination and enhanced THC effects in other outcomes [126].

Pregabalin is a Ca^2+^ channel antagonist used for treating epilepsy and neuropathic pain. Isobolographic analysis demonstrated that combining WIN 55,212-2 with pregabalin exerted synergistic antinociceptive effects in the mouse hot-plate test [127]. Vagus nerve stimulation (VNS) is used as an add-on treatment to patients with drug-resistant epilepsy. Implantation of a vagus nerve stimulator in rats significantly decreased AEA and 2-AG in mesenteric adipose tissue, but increased PEA [128]. Chemical VNS by administration of the peptide hormone cholecystokinin 8 to fasted rats decreased expression of CB_1_ in vagal afferent neurons [129].

### Complementary and alternative medicine

#### Dietary supplements: PUFAs

Polyunsaturated fatty acids (PUFAs) play fundamental roles in many cellular and multicellular processes, including inflammation, immunity, and neurotransmission. They must be obtained through diet, and a proper balance between omega-6 (ω-6) PUFAs and ω-3 PUFAs is essential. The typical Western diet contains a surfeit of ω-6s and a deficiency of ω-3s [130].

Arachidonic acid (AA) is the archetypical ω-6, with 20 carbons and four double bonds (20:4ω-6). Some of its metabolites cause chronic diseases seen in Western populations: prostaglandins cause pain and swelling, and leukotrienes cause bronchoconstriction and asthma. The inflammatory metabolites of AA are countered by dietary ω-3s. The two best-known ω-3s are eicosapentaenoic acid (EPA, 20:5ω-3) and docosahexaenoic acid (DHA, 22:6ω-3).

eCBs are derived from AA (see Figure 2). Several preclinical studies showed that dietary supplementation with AA increased serum levels of AEA and 2-AG, summarized in Table 1. Although we clearly need AA to biosynthesize eCBs, *excessive* levels of AA, administered chronically, may lead to excessive levels of eCBs. This in turn may lead to desensitized and downregulated CB_1_ and CB_2_ receptors. Linoleic acid, an 18:2ω-6 PUFA, is converted into AA, and it elevated 2-AG and AEA levels and induces obesity in mice [131].

**Table 1:** Effects of PUFA supplementation upon eCB levels.

Supplemented PUFA	assay; result compared to unsupplemented controls^1^	reference
DHA+AA	*in vivo* piglets, whole brain homogenates; ↑AEA, ≈2-AG	[137]
AA	*in vivo* mice, whole brain homogenates; ↑AEA	[137]
DHA	*in vivo* mice, whole brain homogenates; ↓2-AG	[325]
AA	*in vivo* mice, whole brain homogenates; ↑2-AG	[325]
DHA	*in vitro* mouse 3T3-F442A adipocytes; ↓2-AG, ↓AEA	[326]
AA	*in vitro* mouse 3T3-F442A adipocytes; ↑2-AG	[326]
DHA+EPA	*in vivo* rats, whole brain homogenates; ≈AEA, ≈2-AG	[327]
or AA	*in vivo* rats, jejunum homogenates; ↑AEA, ↑2-AG	
DHA+EPA	*in vivo* Zucker rats, visceral adipose tissue; ↓↓2-AG, ↓AEA	[142]
DHA+EPA	*in vivo* Zucker rats, whole brain homogenates; ↓2-AG, ≈AEA	[143]
DHA+EPA	*in vivo* rats; serum: ↓↓AEA, ↓2-AG; brain: ↓AEA, ≈2-AG	[133]
DHA+EPA	*in vivo* obese humans; serum: ↓2-AG, ≈AEA	[144]
DHA+EPA	*in vivo* mice; liver: ↓AEA, ≈2-AG; brain: ↓AEA	[131]

1 ↑, increase; ↓, decrease; ≈, no change;

Dietary supplementation with ω-3s predictably increased the concentration of EPA and/or DHA in tissues, cells, and plasma, and decreased the relative concentration of AA in tissues, cells, and plasma [132], [133]. ω-3 supplementation also decreased AEA and 2-AG in tissues, cells, and plasma (Table 1). However, the effects of ω-3 supplementation are nuanced and complex:

Piscitelli *et al.* [134] fed mice a high-fat diet (cholesterol and saturated fatty acids) with little AA. This diet caused a *decrease* in AEA and 2-AG in the liver. Supplementing that diet with DHA and EPA *increased* AEA and 2-AG in the liver. In contrast, the high-fat diet increased AEA and 2-AG in muscle tissue, and supplementation with krill oil *decreased* AEA and 2-AG. Similar trends were seen in heart, kidneys and white adipose tissue.

Adequate levels of dietary ω-3s are required for proper eCB signaling. Mice supplemented with ω-3s, compared to mice on a control diet, expressed greater levels of CB_1_ and CB_2_ mRNA. Mice supplemented with ω-3s also expressed greater levels of eCB synthetic enzymes—NAPE-PLD, DAGLα, and DAGβ [132]. Supplementation with ω-3s also modulated the concentrations of “entourage compounds” such as PEA and OEA [133], [134].

In apparent contrast with the above findings, Lafourcade *et al.* [135] showed that ω-3 *deficiency* abolished eCB-mediated neuronal functions. They reasoned that lifelong ω-3 deficiency causes chronically elevated eCB levels within brain synapses, which leads to CB_1_ desensitization. They tested a rodent model of depression-like behavior (the forced-swim test), and ω-3-deficient mice performed like CB_1_^−/−^ knockout mice. The administration of WIN55212-2 did not change their behavior, whereas in ω-3-rich mice, WIN55212-2 imparted typical cannabimimetic effects. Larrieu *et al.* [136] demonstrated depressive-like symptoms in ω-3-deficient mice compared to mice fed an ω-3 enriched diet. They used the forced-swim test as well as the more valid open-field and social-investigation tests. Mice deficient in ω-3 showed impairment in the CB_1_ signaling pathway—ERK1/2 phosphorylation in the hippocampus was reduced after treatment with WIN55212-2, and the antianxiety effects of WIN55212-2 were absent in ω-3-deficient mice.

ω-3 PUFAs may impact the eCB system via a second mechanism: eCB biosynthetic enzymes readily accept ω-3s as substrates. An ω-3-rich diet markedly elevated the *N*-acyl-ethanolamide metabolite of DHA, called DHEA, the *N*-acyl-ethanolamide metabolite of EPA, called EPEA, and the *sn*-2-glycerol-ester metabolite of EPA, called 2-EPG [133], [137]. FAAH catabolized DHEA [138], [139]. DHEA and EPEA act as eCBs: DHEA and EPEA showed high binding affinity for CB_1_ (*K*_i_ = 124 and 55 nM respectively) and acted as partial agonists [139]. Their affinity nearly equals that of AEA—a meta-analysis of affinity studies using the same binding assay (mouse brain, [^3^H]CP55940 displacement, presence of PMSF) produced a modal *K*_i_ value of 61 nM for AEA [22]. DHEA, *aka* synaptamide, stimulates neurite growth and synaptogenesis in developing hippocampal neurons [140].

In natural fish oil, DHA and EPA are esterified in triacylglycerides (TAG), whereas in many fish oil capsules, DHA and EPA are esterified in EE (ethyl-ester) or TAG (rTAG). Krill oil contains DHA and EPA esterified in phospholipids, primarily phosphatidylcholines, which may improve their bioavailability; furthermore krill oil contains less AA than fish oil [141]. Batetta *et al.* [142] supplemented the diet of obese Zucker rats with fish oil or krill oil, which contained nearly identical amounts of EPA and DHA. The visceral adipose tissue of krill oil-supplemented rats contained less AEA and 2-AG than fish oil-supplemented rats. In the liver only AEA levels were significantly less. The effects of these dietary sources of DHA and EPA on brain eCB levels were much less pronounced, with krill oil producing only a small decrease of 2-AG levels [143]. The same research group reported similar results in an obese cohort mostly composed by women: krill oil but not fish oil significantly decreased serum 2-AG levels; no significant changes were seen in normo-weight subjects [144]. In a yet unpublished study, one of us observed that in obese men, dietary krill oil reduced plasma AEA levels and concomitantly counteracted hypertriglyceridemia (Di Marzo, unpublished data).

In summary, dietary ω-3s seem to act as homeostatic regulators of the eCB system. In obese rodents fed a high-AA diet, ω-3s significantly decrease eCBs, especially 2-AG, particularly in tissues that become dysregulated, such as adipose and liver tissues. Plasma eCB levels are reduced by krill oil also in obese humans. Little change in eCB levels are seen in normo-weight individuals not fed a high ω-6 diet, and dietary ω-3s are required for proper eCB signaling.

#### Dietary supplements: Probiotics

“Probiotics” are endosymbiotic microorganisms that confer a health benefit upon their human hosts. Probiotics occur in fermented foods, such as yogurt and kimchi. The best known organisms are *Lactobacillus acidophilus* and *Bifidobacterium* species. “Prebiotics” such as oligofructose are carbohydrates that serve as substrates for probiotic organisms. Human intestinal epithelial cells incubated with *L. acidophilus* produced more CB_2_ mRNA [145]. Feeding *L. acidophilus* to mice and rats increased the expression of CB_2_ mRNA in colonic epithelial cells. Lastly, mice fed *L. acidophilus* showed less pain behavior following colonic distension with butyrate than control mice, an effect reversed by the CB_2_ antagonist AM630 [145].

Probiotics and prebiotics also modulate CB_1_ expression. Acute probiotic treatment with *Enterococcus faecium* upregulated CB_1_ mRNA in *Solea solea* [146]. Pathologically obese *ob/ob* mice expressed elevated levels of colon CB_1_ mRNA [147]. When fed prebiotics such as oligofructose, they expressed less CB_1_ mRNA, produced less AEA (due to increased FAAH mRNA expression in adipose tissue), and gained less fat mass.

#### Other dietary considerations

A natural phosphate derivative of vitamin E, α-tocopheryl phosphate (α-TP), is a common constituent in plant and animal tissues. Although α-TP does not bind to CB_1_, it modulates synaptic transmission in rodent hippocampus slices, an effect blocked by the CB_1_ antagonist AM251 [148].

Human breast milk contains small amounts of AEA and high levels of 2-AG, but the biological significance of this is not known [149]. The oral administration of AEA (300 mg/kg), OEA (200 mg/kg) and especially 2-AG (400 mg/kg) in rats produces calming properties [150]. Mouse breast milk also contains eCBs, and when newborn mice are fed the CB_1_ antagonist SR141716A, they stop suckling and die [151].

Pesticides such as chlorpyrifos and diazinon alter normal eCB system function [152], [153]. We hypothesize that eating organic foods lacking pesticide residues may promote eCB homeostasis. Piperonyl butoxide, which is a synergist added to insecticides such as pyrethrum, is an efficacious but low-potency antagonist of CB_1_ [154]. Phthalates are plasticizers added to water bottles, tin cans, food packaging, and even the enteric coating of pharmaceutical pills. Phthalates may act as endocrine disruptors and carcinogens. They also block CB_1_ as allosteric antagonists [155].

#### Herbal remedies

Some plants besides *Cannabis* produce vaguely cannabimimetic effects. Copal incense, extracted from *Protium* species (same plant family as *Boswellia*) contains a pentacyclic triterpene with high affinity for CB_1_ and CB_2_ [156]. Absinthe contains thujone, a constituent of wormwood, *Artemisia absinthium*. Thujone has weak affinity for CB_1_ [157]. Pristimerin, an alkaloid found in khat, *Catha edulis*, acts as a potent inhibitor of MAGL (IC_50_ = 93 nM) and causes an elevation of 2-AG levels in rat cortical neurons [158]. Salvinorin A in *Salvia divinorum* produces CB_1_-mediated effects in the gastrointestinal tract of rodents. Salvinorin A primarily acts as a kappa-opioid receptor agonist and is inactive as a ligand for CB_1_ and CB_2_ [159]; it may interact with a putative CB_1_-kappa-opioid receptor heterodimer [160].

Flavonoids such as biochanin A (from red clover, *Trifolium pratense*), genistein (from soybean, *Glycine max*), and kaempferol (from tea, *Camelia sinensis*, and many other plants) exert modest inhibition of FAAH in the low micromolar range [161]. Cyanidin and delphinidin, two anthocyanidins found in a wide range of plants, have micromolar affinities for CB_1_ [162]. Epigallocatechin-3-O-gallate, the most abundant catechin in tea, also has micromolar affinities for CB_1_ [163].

Yangonin, a kavalactone extracted from kava, *Piper methysticum*, exhibits affinity for CB_1_ with a *K*_i_ = 0.72 µM [164]. Curcumin, extracted from curry powders, elevates eCB levels and brain nerve growth factor (NGF) in a brain region-specific fashion, and pretreatment with CB_1_ antagonist AM4113 blocks this effect [165]. A study suggested that curcumin and resveratrol could bind to CB_1_, but the study was retracted [166].

Compounds with phytocannabinoid-like moieties have been extracted from legumes [167], [168], *Helichrysum* [169], *Rhododendron* sp. [170], liverworts [171], [172], and fungi [173]–[175]. Falcarinol is a skin irritant found in several plants that causes contact dermatitis. It covalently binds with the CB_1_ receptor, causing potent inverse agonistic and pro-inflammatory effects in human skin [176].

Higher plants (angiosperms and gymnosperms) produce PUFAs with acyl tails limited to 18 carbons in length [177]. Hence reports of PUFAs in plants with longer acyl tails, such as AA, AEA, and 2-AG are controversial. Di Tomaso *et al.* [178] detected AEA in chocolate and cocoa powder derived from *Theobroma cacao*. A subsequent study showed very little, if any, AEA in cocoa powder [149]. Nakane *et al.* [179] reportedly extracted sciadonic acid (20:3ω-6) from seeds of a pine tree, *Sciadopitys verticillata*. This analog of 2-AG exhibited cannabimimetic activity in NG108-15 cells expressing CB_1_.

Unlike higher plants, non-vascular plants such as club mosses, mosses, and algae express Δ^6^-elongase enzymes, so they are capable of producing PUFAs with longer acyl tails [177]. Semiplenamide A, an AEA-like PUFA isolated from a blue-green alga, *Lyngbya semiplena*, has micromolar affinity for CB_1_ and also blocks the AEA transporter, thereby inhibiting AEA breakdown [180]. Grenadamide, a PUFA in *Lyngbya majuscula*, has micromolar affinities for CB_1_ [181]. Soderstrom *et al.* [182] extracted but did not identify an eCB-like compound from *L. majuscula*. Soderstrom also extracted a dozen eCB-like PUFAs from unidentified green algae (Chlorophyta), the brown alga *Laminaria angustata*, and the sponge *Mycale micracanthoxea*.

Some plant ligands bind to CB_2_ and modulate the immune system, but have no affinity for CB_1_ and do not elicit psychoactivity. Alkamides from *Echinacea* species bind to CB_2_ with nanomolar affinity, and act as CB_2_ agonists with immunomodulatory effects [183]. Several constituents from *E. purpurea* root and herb produce synergistic, pleiotropic effects—they bind to CB_2_ as well as inhibit AEA uptake [184]. Other constituents from *Echinacea purpurea* act as weak CB_1_ antagonists [185].

The principal terpenoid in black pepper, (E)-β-caryophyllene (BCP), binds to CB_2_ with nanomolar affinity and acts as an agonist. Its anti-inflammatory effects are reduced in CB_2_ knockout mice [186]. The protective effects of BCP on colitis in mice are reversed by the CB_2_ antagonist AM630 [187]. The protective effects of BCP on cisplatin-induced nephrotoxicity in mice are absent in CB_2_ knockout mice [188]. Lastly, the antinociceptive effect of BCP in mice is prevented by pretreatment with AM630 [189].

Rutamarin in *Ruta graveolens* has micromolar affinity for CB_2_ [190]. An unidentified constituent in noni fruit, *Morinda citrifolia*, shows weak affinity for CB_2_ [191]. The aromatic resin extracted from mastic, *Pistacia lentiscus*, contains an essential oil (EO) rich with monoterpenoids and sesquiterpenoids. Rats fed mastic EO showed higher plasma levels of DHA, EPA, PEA, and OEA than control rats, with no change in AEA or 2-AG [192].

Shellfish are not herbal remedies, but they have been used medicinally. AEA and/or 2-AG have been isolated from the mussel *Mytilus galloprovincialis*, the clam *Tapes dicussatus*, the oyster *Crassosterea* sp. [193], the sea urchin *Paracentrotus lividus* [194], and the sea squirt *Ciona intestinalis* [195].

#### Mind and body medicine: chronic stress

Chronic or repeated stress results in a chronic elevation of endogenous corticosterone via the hypothalamic-pituitary-adrenocortical (HPA) axis. Chronic stress (repeated restraint) reduced AEA levels throughout the corticolimbic stress circuit in rodents [99], [196], [197]. In contrast, 2-AG levels decrease or increase, depending upon the nature of the stressor: Hill *et al.* [198] found reduced 2-AG content within rat hippocampus following the CUS protocol. But in the hypothalamus and midbrain, 2-AG increased in the same testing paradigm [99]. Elevations in 2-AG appear after chronic restraint stress within the amygdala [196], [199], hypothalamus [200], and medial prefrontal cortex [58].

CB_1_ expression decreased in rat hippocampus following the CUS protocol [198], whereas CB_1_ expression increased in the prefrontal cortex in the same testing paradigm [99]. The same paradigm decreased hippocampal CB_1_ expression in male rats, but increased CB_1_ expression in female rats [201]. Social isolation stress decreased CB_1_ density in the supraoptic nucleus of rats [202]. Immobilization/acoustic stress increased CB_1_ mRNA and protein expression in the prefrontal cortex of mice [203]. A chronic mild stress protocol (subjecting rats to cage soiling with water, group housing in a confined space, water and/or food deprivation, intermittent lighting, reversal of light/dark cycle, cage tilting to 45°, exposure to loud white noise and strobe lights) increased CB_1_ mRNA in the prefrontal cortex and decreased CB_1_ in the midbrain [204].

Adult rats exposed to chronic restraint stress increased CB_1_ binding of [^3^H]CP55,940 in the prefrontal cortex (PFC) with a decrease in the hippocampus. A 40-day recovery period resulted in normalization of CB_1_ in the PFC, and a pronounced upregulation of CB_1_ density in the hippocampus, possibly indicative of a rebound effect. Adolescent rats did not show any change in hippocampal CB_1_ density, but exhibited an upregulation in both the PFC and amygdala. They also exhibited a rebound in the hippocampus after 40 days [205].

Chronic water avoidance stress in male rats increased serum corticosterone levels and visceral hyperalgesia in response to colorectal distension, accompanied by increased AEA, decreased CB_1_ expression, and increased TRPV1 expression in the dorsal root ganglia [62]. Co-treatment with the corticoid receptor antagonist RU-486 prevented these changes [206]. Seven daily sessions of social defeat stress in mice decreased AEA levels in the hypothalamus and hippocampus, but not in the striatum or the frontal cortex; 2-AG levels increased after the last, but not the first, session in the hypothalamus, hippocampus, and frontal cortex [207]. Fear expression after the sessions was prolonged in mice receiving rimonabant and in CB_1_^−/−^ knockouts. Conditional knockouts lacking CB_1_ in two defined neuronal subpopulations—glutamatergic neurons and GABAergic neurons—indicated that the former CB_1_ subpopulation was responsible for the fear responses.

Electrophysiological studies confirm the effects of chronic stress upon the eCB system: Chronic social defeat stress in mice (exposure to aggression) impaired GABAergic synapse sensitivity to eCBs (probably 2-AG) mobilized by group I metabotropic glutamate receptor stimulation [208]. The CUS protocol attenuated eCB-mediated DSE, LTD, and depression of field excitatory postsynaptic potentials [96]. Chronic restraint stress attenuated eCB-mediated DSI in rat hippocampus [209]. These chronic stressors also desensitized CB_1_ to exogenous cannabinoids: they reduced electrophysiological responses to HU210 in mouse striatum [208], and to WIN55,212-2 in mouse striatum [96]. Chronic immobilization stress in rats impaired retrograde eCB signaling at GABAergic synapses, and a functional downregulation of CB_1_ in the paraventricular nucleus of the hypothalamus [210].

*Acute* restraint challenge in rats induces corticosterone release in the paraventricular nucleus of the hypothalamus (PVN). This is inhibited by dexamethasone, a response blocked by the CB_1_ antagonist AM251—suggesting that fast feedback requires local release of eCBs. Indeed, PVN content of 2-AG is elevated by the restraint challenge [200].

Acute footshock stress increased 2-AG and AEA levels in the periaqueductal gray and contributed to stress-induced analgesia (SIA) in male rats. SIA enhancement by a MAGL inhibitor and not by a FAAH inhibitor indicated that 2-AG was the primary eCB responsible for SIA [211]. SIA was modulated via CB_1_ receptors in the basolateral nucleus of the amygdala (BLA); microinjection of SR141716A into the BLA suppressed SIA [212]. Glucocorticoid enhancement of memory consolidation in the acute footshock stress is dependent upon CB_1_ activation in male rats; WIN55,212-2 infused into the amygdala enhances memory in an inhibitory avoidance apparatus, and AM251 impairs the response [213]. Acute handling stress in male newts increased serum cortisol levels and induced behavioral changes (less sexual behavior); the latter was blocked by a cannabinoid antagonist, AM281, indicating dependence upon CB_1_ activation [214].

Acute restraint stress in *male* rats increases hippocampal content of 2-AG and enhanced eCB-dependent modulation of GABA release measured by whole-cell voltage clamp of inhibitory post-synaptic currents (IPSCs) in hippocampal CA1 cells [215]. Responses in *female* rats are much more complex, because eCB levels fluctuate across the estrous cycle [216]. The eCB system has been implicated in cycle-dependent changes in pressure pain thresholds in human females [217].

In summary, chronic stress impairs the eCB system, via decreased levels of AEA and 2-AG. Changes in CB_1_ expression are more labile. Stress management may reverse the effects of chronic stress on eCB signaling, although few studies exploring this possibility have been performed to date. Clinical anecdotes suggests that stress-reduction techniques, such as meditation, yoga, and deep breathing exercises impart mild cannabimimetic effects [218].

Rossi *et al.* [208] found that mice given access to a running wheel recovered their chronic stress-induced synaptic defects. Accordingly, social play in rats increased CB_1_ phosphorylation (a marker of CB_1_ activation) in the amygdala and enhanced AEA levels in the amygdala and nucleus accumbens [219]. The effects of exercise on the eCB system are elaborated below. Grooming behavior, which is a stress-reduction behavior in rodents, increased in response to SR141716A administration [220].

#### Mind and body medicine: acupuncture

Acupuncture reduced stress-related behavior (from maternal separation in rats) and normalized HPA-induced corticosterone release [221]. Electroacupunture (EA) reduced thermal hyperalgesia and mechanical allodynia induced by an injection of complete Freund's adjuvant into rat paws. EA increased AEA levels in skin tissue. The antinociceptive effects of EA were attenuated by the CB_2_ antagonist AM630, but not by the CB_1_ antagonist AM251 [222]. Moreover, EA upregulated the expression of CB_2_ receptors in skin tissues [223]. It appears likely that CB_2_ activation in the skin stimulates the release of β-endorphin, which then acts on peripheral μ-opioid receptors to inhibit nociception [224].

However, CB_1_ may play a role in the *central* effects of EA: rats treated with EA showed reduced GABA levels in the ventrolateral periaqueductal gray, an effect reversed by CB_1_ blockade with AM251 [225]. Enhanced activation of epsilon protein kinase C in rat brain by EA was reversed by CB_1_ blockade with AM251 and not by CB_2_ blockade with AM630 [226].

#### Mind and body medicine: body-based practices

Massage and osteopathic manipulation of asymptomatic participants increased serum AEA 168% over pretreatment levels; mean OEA levels decreased 27%, and no changes occurred in 2-AG. Participants receiving sham manipulation showed no changes [218]. Osteopathic manipulation of participants with low back pain increased serum PEA 1.6-fold over pretreatment levels, with no change in AEA. Participants receiving sham manipulation showed no changes [227].

### Lifestyle modifications

#### Diet and weight change

Dozens of animal studies and human cohort studies have shown that diets rich in fats and sugars alter levels of AEA, 2-AG, their metabolic enzymes, and CB_1_. The reverse causality is also true—many studies show that CB_1_ agonists stimulate the consumption of fat and sugar. The rewarding properties of palatable foods are attenuated by CB_1_ blockade and in CB_1_^−/−^ knockouts. Stimulation of feeding behavior by CB_1_ agonists occurs across the phylogenetic scale, from humans to *Hydra*, although there is no molecular evidence for CB_1_ orthologs in invertebrates other than the boneless chordates *Ciona intestinalis* and *Branchiostoma floridae*. Reviews on this topic are available [7], [228], [229], which we do not intend to duplicate here.

Upregulation of the eCB system in obese humans seems to be driven by excessive production of eCBs in several peripheral tissues such as visceral adipose tissue, liver, pancreas, and skeletal muscle. Differences arise between central (intra-abdominal) adipocytes versus peripheral (subcutaneous) adipocytes, with additional variations due to gender, age, and genetic polymorphisms in metabolic enzymes. Visceral adiposity particularly correlates with elevated levels of 2-AG in blood plasma [230]. Increases in circulating eCBs likely reflect spillover from adipose tissues and liver parenchyma, where CB_1_ activation promotes *de novo* lipogenesis and reduces insulin sensitivity, respectively. In mice with diet-induced obesity, CB_1_ mRNA and protein levels increased in the hippocampus, compared to lean controls [231]. Furthermore, hippocampal slices from obese mice showed increased CB_1_ functionality, with no sign of CB_1_ desensitization. We find it surprising that sustained elevations of eCB ligands do not result in CB_1_ downregulation. This may be due to the fact that such elevations are not as dramatic as those caused, for example, by chronic MAGL inhibition. The lack of downregulation may contribute to the hedonic aspects of overeating, and influence cognitive processes.

Weight loss by caloric restriction or fasting predictably modulates the eCB system. Animal studies have demonstrated the complexities arising in adipose tissue versus the central nervous system (Table 2). In human studies, weight loss from caloric restriction has produced conflicting results. Engeli *et al.* [232] measured CB_1_ and FAAH gene expression, and serum AEA and 2-AG, in obese postmenopausal women. They reported no changes after 5% weight loss from caloric restriction. Bennetzen *et al.* [233] analyzed a younger population of obese men and women; a 10–12% weight loss resulted in elevated 2-AG levels in gluteal adipose tissues, with no change in AEA levels. Weight loss increased CB_1_ mRNA in abdominal adipose tissues but decreased CB_1_ mRNA in gluteal adipose tissues.

**Table 2:** Effects of short- and long-term caloric restriction upon the brain eCB system in animal studies.

species, exercise	measure	reference
rats administered leptin	leptin (appetite-reducing hormone) decreases hypothalamic AEA and 2-AG levels	[6]
rats fasted	fasting for 24 h increased AEA and 2-AG in limbic forebrain and 2-AG in hypothalamus;	[328]
mice fasted	time-dependent effects: short-term fasting (24 h) increased hypothalamic 2-AG; long-term fasting (12 d) decreased hypothalamic 2-AG	[329]
goldfish fasted	food restriction decreased CB_1_ mRNA in the forebrain and increased AEA levels in the telencephalon, two effects reversed by refeeding	[330], [331]
rats after gastric bypass	weight loss after Roux-en-Y gastric bypass surgery decreased AEA and with no change in 2-AG levels in skeletal muscle	[332]
Zucker obese rats fasted	fasting decreased CB_1_ mRNA in brainstem but not in hypothalamic nuclei	[333]

In centrally obese men, decreased plasma AEA and 2-AG levels accompanied a weight loss intervention consisting of both caloric restriction and exercise. Only 2-AG levels correlated with decreased visceral adipose tissue, plasma triglycerides and insulin resistance, and improved HDL-cholesterol levels [234]. However, the influence of caloric restriction and exercise separately was not analyzed in this study. You *et al.* [235] measured CB_1_ and FAAH mRNA in subcutaneous abdominal and gluteal adipose tissue in overweight or obese postmenopausal women. Caloric restriction resulted in 11% weight loss, which led to a reduction in gluteal CB_1_ and FAAH gene expression but no significant changes in abdominal adipose tissue. You and associates also tested the effects of exercise, see below. A 12-week hospital-based weight loss program (moderate caloric restriction along with counseling by dieticians and physical activity teachers) resulted in a mean weight loss of 9.5% and a significant reduction in salivary AEA levels, while salivary 2-AG, OEA and PEA did not significantly change [236].

In summary, increased food intake, adiposity, and elevated levels of AEA and 2-AG apparently spiral in a feed-forward mechanism. Weight loss from caloric restriction breaks the cycle, possibly by reducing CB_1_ expression and reducing eCB levels.

#### Exercise

Rodent studies have shown that exercise modulates the eCB system (Table 3). The results of these studies show a critical difference between short-term, voluntary exercises (*e.g.*, wheel running) and long-term, coerced exercise (forced swimming, treadmills). Although both types of exercise regimens increased eCB ligand concentrations, only long-term-forced exercise led to sustained elevations of eCBs, and predictable CB_1_ downregulation.

**Table 3:** Effects of exercise upon the eCB system in rodent studies.

species, exercise	measure	reference
rats, forced swimming for 1 h/d×6 months	decreased CB_1_ antibody expression in adipocytes	[334]
rats, voluntary wheelrunning, 24 h	running reversed chronic stress-induced deficits in GABAergic synapses to CB_1_ stimulation by eCBs and HU210	[208]
mice, voluntary wheel running, 42 d	running rescued the sensitivity of striatal GABA synapses to CB_1_ stimulation downregulated by EAE induction	[335]
mice, voluntary wheel running for 15 d	sensitivity of striatal GABA synapses to CB_1_ stimulation increased	[336]
rats, forced treadmill running for 40 d	reduced CB_1_ expression in the striatum and hippocampus	[337]
rats, voluntary wheel running for 8 d	increased CB_1_ expression in the hippocampus, increased CB_1_-mediated GTPγS binding, and increased AEA content in hippocampus	[338]
mice, voluntary wheel running for 10 d	increased CB_1_ expression in the hippocampus	[321]
rats, forced treadmill running for 40 d	no change in gene expression of CB_1_, CB_2_, or FAAH in liver	[339]

In humans, serum AEA levels doubled over baseline in male subjects after ≥30 min running, and increased significantly in male subjects after biking. Serum 2-AG levels did not significantly increase [237]. Heyman *et al.* [238] reported similar findings in male cyclists—serum AEA levels increased significantly during exercise, whereas 2-AG concentrations remained stable. AEA levels increased incrementally at 55% maximum work output, at 75% W_max_, and during a 15 min recovery period. Beta-endorphin levels exhibited a different trajectory—they did not increase until the 75% W_max_ stage, and dropped significantly during the recovery period.

Feuerecker *et al.* [239] measured the effects of physical exercise in aerobically-trained male subjects. Strenuous hiking at high altitudes (up to 3196 m) significantly increased serum AEA levels over baseline. Strenuous hiking at low altitudes also increased AEA level, but to a lesser extent. In a small cadre of overweight or obese middle-aged women, 20 weeks of moderate-intensity aerobic exercise (CRM) or vigorous-intensity aerobic exercise (CRV) did not change CB_1_ or FAAH gene expression [235]. However, combining data from the two groups (CRM+CRV) showed a decrease in FAAH mRNA in abdominal adipose tissue, compared to a control group that participated solely in caloric restriction. The CRM and CRV groups showed a slight increase in CB_1_ mRNA expression in gluteal adipose tissue over baseline, whereas the control group that only participated in caloric restriction showed a significant decrease in CB_1_ mRNA.

Raichlen *et al.*, [240] measured circulating eCBs in humans and dogs (cursorial mammals) and ferrets (a non-cursorial mammal) before and after treadmill exercise to test the hypothesis that neurobiological rewards are linked to high-intensity exercise in cursorial mammals. The authors showed that humans and dogs share significantly increased exercise-induced eCB signaling following high-intensity endurance running, whereas eCB signaling did not significantly increase following low-intensity walking, nor did it increase in the non-cursorial ferrets following exercise at any intensity. The same research group showed that serum AEA levels in male and female runners significantly increased after 30 minutes of moderately intense treadmill running (70–80% age-adjusted maximum heart rate), and not after very high or very low intensity exercises [240], [241].

In summary, medium- to high-intensity voluntary exercise in cursorial mammals, including humans, increases eCB signaling, via increased serum AEA levels (but not 2-AG), and possibly increased CB_1_ expression. “Runner's high” may be an eCB-induced reward for exercise.

#### Alcohol

*Acute* administration of a high dose of ethanol in rats decreased AEA levels in brain, serum, and adipose tissue; PEA also decreased in the brain. AEA decrease was associated with inhibition of AEA release and no change in NAPE-PLD or FAAH hydrolysis [242]. However, exposing *ex vivo* murine hippocampal neuron cultures to lower doses of ethanol increased AEA and 2-AG release [243]. This increase led to reduced presynaptic glutamate release in neuron cultures, which was blocked by SR141716A. There was no change in CB_1_ density.

Electrophysiological studies of anesthetized rats showed that alcohol enhanced eCB signaling in mesolimbic circuits [244]. This effect was blocked by SR141716A, and increased by the FAAH inhibitor URB597—indicating AEA involvement. Another study by the same group showed parallel responses in rat amygdala. The downregulation of amygdala CB_1_ with chronic WIN55212-2 blunted the response to alcohol [245].

*Ex vivo* exposure of rat striatal slices showed ethanol shifts synaptic plasticity from LTP to eCB-mediated LTDI. Ethanol-enhanced LTDI was blocked by the CB_1_ antagonist AM251 [246]. The same group showed that ethanol modulated eCB-mediated striatal plasticity in a synapse-specific manner. Ethanol prevented CB_1_-dependent long-lasting disinhibition (DLL) in the dorsolateral striatum [247]. Furthermore, the study showed that LTDI by an exogenous cannabinoid, WIN55,212-2, was actually prevented by ethanol.

*Chronic* ethanol treatment decreased CB_1_ density and decreased cannabinoid-stimulated [^35^S]GTP*γ*S activation in various animal models [248]–[251]. One study of chronic ethanol did not alter CB_1_ binding of [^3^H]CP55,940 or CB_1_ mRNA levels in rat brain homogenates [68]. Short-term chronic exposure (72 hours) of ethanol vapor in mice increased CB_1_ density in the cortex, hippocampus, striatum and cerebellum, with downregulation of CB_1_ receptor-stimulated [^35^S]GTP*γ*S binding [252]. The effects of chronic ethanol treatment upon eCB levels in various *in vitro* and animal models are shown in Table 4.

**Table 4:** Effects of chronic or subchronic ethanol upon eCB levels.

species, assay	result compared to controls^1^	reference
human neuroblastoma cell line	↑ [^3^H]AEA	[340]
rat cerebellar granule neurons	↑ [^3^H]2-AG	[341]
rat, oral administration	↑ AEA limbic forebrain, ↓ AEA+2-AG midbrain	[258]
rat cerebellar granule neurons	↑ [^3^H]AEA via ↓ AEA transport and ≈FAAH	[342]
mouse, ethanol vapor inhalation	↑ AEA cortex via ↓ FAAH	[252]

1 ↑, increase; ↓, decrease; ≈, no change; assay; result compared to unsupplemented controls.

Vinod *et al.* [251] compared alcohol-preferring (aP) and alcohol-non preferring (NaP) rats, a pair of rat lines selectively bred for opposite alcohol preference. CB_1_ receptor density, CB_1_ receptor-stimulated [^35^S]GTP*γ*S coupling, and levels of AEA and 2-AG were higher in the brains of alcohol-naive aP compared to NaP rats. Ethanol consumption in aP rats decreased CB_1_ receptor-stimulated [^35^S]GTP*γ*S binding after 10 days, and moreso after 60 days. 2-AG levels elevated after 10 days, and both 2-AG and AEA levels increased after 60 days; FAAH levels decreased with no change in MAGL. Ethanol withdrawl upregulated [^35^S]GTP*γ*S binding.

A rat model of binge drinking—serial cycles of ethanol intoxication and withdrawal—increased CB_1_ mRNA in the prefrontal cortex [253]. Another study of serial cycles in rats showed a transient decrease in hippocampal CB_1_ mRNA and protein levels (two days after cessation of cycles), followed by a long term up-regulation in CB_1_ mRNA and protein, 40 days after cessation of cycles. Serial cycles increased 2-AG in the hippocampus, two days and 40 days after cessation of cycles; AEA increased only at 40 days [254].

An electrophysiological study of intermittent ethanol consumption in rats showed depression of CB_1_-dependent long-lasting disinhibition (DLL) in excised slices of the dorsolateral striatum [255]. Furthermore, the study showed that LTDI by an exogenous cannabinoid, WIN55,212-2, was prevented by intermittent ethanol consumption.

A human clinical trial assigned 55 adults to one of three groups—drinking either 250 ml of red wine, grape juice, or plain water. Within 10 minutes, the consumption of a moderate amount of alcohol reduces plasma AEA and 2-AG concentrations, whereas an equal volume of grape juice did not affect plasma eCBs. Interestingly, plain water reduced 2-AG concentrations without affecting AEA [256].

Alcoholics who died of natural causes or motor vehicle accidents expressed decreased CB_1_ densities in the ventral striatum, decreased CP55,940-stimulated [^35^S]GTP*γ*S binding, and decreased FAAH activity, compared to controls [257]. Alcoholics who died of suicide in the same study had increased CB_1_ densities, increased CB_1_ receptor-stimulated G(i/o) protein activation, and decreased FAAH activity, compared to controls. Lehtonen *et al.* (2010) measured eCB levels in post-mortem brains of Cloninger type 1 and type 2 alcoholics. Type 1 alcoholics had lower levels of AEA than controls in the nucleus accumbens (NAcc), anterior cingulate cortex, and frontal cortex. PEA, OEA, and 2-AG were unchanged. They also showed dopaminergic deficiencies in the NAcc, suggesting a compensatory mechanism one direction or the other. Type 2 alcoholics produced slightly higher eCB levels than controls, but not significantly.

In summary, acute ethanol may enhance endogenous eCB release and eCB signaling, although it varies by brain area and synapse, and this complexity requires further testing. Two studies suggest ethanol dampens the effects of the eCB system. Chronic ethanol consumption and binge drinking likely desensitize or downregulate CB_1_ and impair eCB signaling, except perhaps in areas involved in reward and motivation to self-administer this substance of abuse [258].

#### Nicotine

In a human randomized controlled trial, nicotine augmented THC-induced “high” and heart rate [259]. In rodent behavioral studies, *acute* nicotine augmented THC discrimination and THC-induced hypothermia, antinociception, locomotor inactivity, anxiolysis, and place aversion [260]–[264]. Nicotine-potentiated THC discrimination was blocked by rimonabant and URB-597 (a FAAH inhibitor), suggesting nicotine potentiation is mediated by the release of AEA acting at CB_1_ [263]. CB_2_ is also involved—the CB_2_–selective agonist JWH133 induced antinociception in the mouse formalin test, and this effect was potentiated by nicotine [265]. Acute nicotine elicited marked increases in AEA in the amygdala, hypothalamus, and prefrontal cortex but decreased levels in the hippocampus; variations in 2-AG were less pronounced [266].

In a contrary study, intracelebellar microinfusion of nicotine attenuated THC-induced ataxia in mice. Microinfusion of synthetic subtype agonists indicated the involvement of α_4_β_2_ but not α_7_ nicotinic receptor subtypes [267]. Buczynski *et al.* [268] compared volitional self-administration (SA) versus forced nicotine exposure (FA) in the ventral tegmental area using *in vivo* microdialysis. SA but not FA increased AEA; both SA and FA increased 2-AG; these subtle changes were not seen in corresponding bulk brain tissue analysis of eCBs. Acute nicotine enhanced THC-induced c-Fos expression in various brain regions [264].

*Chronic* nicotine increased AEA levels in the limbic forebrain and increased AEA and 2-AG contents in rat brainstem, but decreased AEA and/or 2-AG contents in the hippocampus, the striatum and the cerebral cortex [258]. Chronic nicotine increased CB_1_ density in the prelimbic prefrontal cortex, ventral tegmental area, and the hippocampus [269]. Seven days of nicotine exposure increased brain CB_1_ densities in adolescent male rats and sensitized them to the locomotor-decreasing effects of THC and CP55,940 [270]. These changes were not seen in adult male rats. Chronic nicotine inhibited the development of tolerance to antinociceptive and hypothermic effects of THC [264].

Other plant products that exert cholinergic effects, such as calamus, *Acorus calamus*, have been admixed with cannabis to decrease cannabis-induced memory deficits, and “calm and center the effects of marijuana” [42]. Consistent with this, the synthetic cholinergic agent rivastigmine reversed memory deficits in rats induced by the synthetic cannabinoid WIN55,212-2 [271].

#### Caffeine

Co-administering caffeine and cannabis has a long history. Bell [272] claimed that oral administration of hashish with coffee increased the effects of cannabis, and at the same time diminished its duration. He proposed a pharmacokinetic mechanism—coffee promoted more rapid absorption of hashish.

Caffeine and theophylline are antagonists of adenosine receptors. Adenosine receptors are tonically activated by adenosine, their endogenous ligand. Rodent studies indicate that A_1_-subtype adenosine receptors tonically inhibit CB_1_ activity [273]. Thus the antagonism of A_1_ receptors by caffeine and theophylline enhances eCB system function (*e.g.*, activation of CB_1_ by 2-AG). Caffeine potentiated CB_1_-mediated activity stimulated by THC and WIN-55,212 in hippocampus slices [273]. Consistent with this, the simultaneous application of WIN-55,212 plus an A_1_ agonist produced less than additive stimulation of [^35^S]GTP*γ*S binding in mouse cerebellar membranes [274].

In whole animals, however, caffeine's effects are biphasic and vary by dosage and acute versus chronic administration. In humans, the acute administration of caffeine decreases headache pain, but exposure to chronic high doses, ≥300 mg/day, may exacerbate chronic pain [275]. In rabbits, an acute dose of caffeine antagonized THC-induced changes in cortico-hippocampal electroencephalogram recordings [276]. In mice, chronic caffeine at high doses potentiated CB_1_-dependent stimulation by eCBs and HU210 at striatal GABAergic, but not glutamatergic, synapses [277]. A single dose or a subacute dose (one day of caffeine in water) rescued the sensitivity of GABAergic synapses to HU210 in mice exposed to chronic stress.

Chronic caffeine at moderate doses increased THC's effects on short-term memory in mice [278]. Surprisingly, CB_1_ density decreased in the caffeinated mice, measured by [^3^H]SR141716A binding. Cortical and hippocampal tissues also showed a decrease in WIN55,212-2-stimulated [^35^S]GTP*γ*S binding, but this attenuation was not seen in THC-stimulated [^35^S]GTP*γ*S binding. This highlights the fact that caffeine-induced changes observed *in vitro* do not necessarily reflect the effects of caffeine upon integrated brain circuitry *in vivo*. Lastly, acute antagonism of A_1_ with DPCPX did not modulate the effects of THC on short-term memory [278], which further supports our hypothesis that chronic and acute blockade of A_1_ receptors have different functional consequences.

#### Cannabis

*Cannabis* and cannabis products are complex polypharmaceuticals, consisting of THC, cannabidiol (CBD), dozens of minor cannabinoids, as well as terpenoids, flavonoids, and other compounds. Fundamentally, THC mimics AEA and 2-AG by acting as an agonist at CB_1_ and CB_2_ [279]. But rather than simply substituting for AEA and 2-AG, McPartland and Guy [280] proposed that *Cannabis* and its many constituents work, in part, by “kick-starting” the eCB system. The acute administration of THC increased CB_1_ density in rodent brains [281], [282]. Acute upregulation of CB_1_ mRNA continued for up to 14 days in some rat brain regions [283]. Acute THC also increased the sensitivity of CB_1_ to cannabinoids, measured by WIN-55,212-2-stimulated [^35^S]GTP*γ*S binding in rat brains [284]. Lastly, acute THC stimulated AEA biosynthesis [285].

Chronic, high dosing of THC causes a predictable desensitization and downregulation of CB_1_ and CB_2_, accompanied by drug tolerance. Chronic THC decreased CB_1_ density in rodent brains, and dampened cannabinoid-stimulated [^35^S]GTP*γ*S [282], [284], [286], [287]. CB_1_ in different regions of the brain downregulate and desensitize at unequal rates and magnitudes, with greatest decreases in the hippocampus and little or no change in the nucleus accumbens and basolateral amygdala. Chronic THC elicited few changes in AEA or 2-AG levels in rat brains, except for a significant augmentation of AEA levels in the limbic forebrain [288].

Similar results have been reported in two human studies. Villares [289] collected postmortem brain tissues from known cannabis smokers; [^3^H]SR141716A binding and CB_1_ mRNA was downregulated in several brain regions, compared to non-smoking control autopsies. Hirvonen *et al.* [290] employed PET scan imaging in living subjects. The degree of CB_1_ downregulation correlated with years of chronic cannabis smoking. CB_1_ densities returned to normal after four weeks of abstinence. Variable downregulation in different brain regions may explain why frequent users of cannabis develop tolerance to some effects of THC, such as anxiogenesis and cognitive impairment, but not to its euphoric effects [291]. Downregulation is partially epigenetic—the CB_1_ promoter region in chronic marijuana smokers is hypermethylated, reducing CB_1_ mRNA expression levels [292].

THC acts as a partial agonist of CB_1_, compared to synthetic cannabinoids which act as full agonists (Table 5). Partial agonism likely explains why exposure to THC caused half as much CB_1_ desensitization as the full agonist WIN55,212-2 in rat hippocampal neurons [293]. In a study of rat CB_1_ transfected into AtT20 cells, THC caused less downregulation and internalization than WIN55,212-2 or CP-55,940 [294]. In agreement, drug tolerance studies utilizing the behavioral “tetrad” test show that chronic THC caused less tolerance than the full agonist CP-55,940 in mice [295]. In a study of human CB_1_ transfected into *Xenopus* oocytes, the desensitization rate of THC was half that of WIN55,212-2 [296]. However, one [^35^S]GTP*γ*S autoradiography study of rat brains suggested that chronic THC and WIN55,212-2 caused equal desensitization [297]. Another study indicated that THC acts as a full agonist at mouse GABAergic synapses, with efficacy equal to WIN55,212-2, albeit at fairly high concentrations [298].

**Table 5:** Partial agonism of THC at CB_1_, based on assays of cannabinoid-stimulated signal transduction.

full agonist, species and substrate	assay; maximal stimulation by Δ^9^-THC compared to the full agonist	reference
WIN55,212-2rat cerebellar membranes	[^35^S]GTP*γ*S binding; 20%	[284]
WIN55,212-2mouse brain membranes	[^35^S]GTP*γ*S binding; 25%	[343]
CP55,940rat cerebellar membranes	[^35^S]GTP*γ*S binding; 54%	[344]
WIN55,212-2rat hippocampal neurons	patch-clamp Ca++ currents and excitatory postsynaptic currents; 41% and 55%	[299]
HU-210, WIN55,212-2transfected human CB_1_	[^35^S]GTP*γ*S binding; 56% at Gai;, 89% at Gao;	[312]
WIN55,212-2transfected human CB_1_	inwardly rectifying potassium (GIRK) current amplitude, 35%	[296]

If THC is a partial agonist, then THC might functionally antagonize the effects of a full agonist when the two drugs are added together. THC antagonized the effects of WIN55,212-2 in rat brain sections [284], [299], and mouse autaptic hippocampal neurons [300].

The capacity of THC to antagonize a full agonist depends, in part, upon ligand affinity—its ability to occupy and hold the CB_1_ binding site. A meta-analysis of affinity studies calculated a mean *K_i_* = 42.6 nM for THC in rat membranes—much less affinity than that of WIN-55,940, with a *K_d_* = 2.4 nM [22]. This indicates that high concentrations of THC relative to WIN-55,940 are required to antagonize the full agonist. There are species differences—in human membranes, CB_1_ affinity of THC (*K_i_* = 25.1 nM) is much closer to that of WIN-55,940 (*K_d_* = 16.7).

2-AG acts as a full agonist at rodent and human CB_1_ and CB_2_ [296], [301]–[303]. The emetogenic effects of exogenously-administered 2-AG were blocked by THC [304]. THC dampened or occluded eCB-mediated retrograde signaling of CB_1_, presumable mediated by 2-AG [300], [305], [306]. Roloff and Thayer [307] demonstrated another complexity in the relationship between THC and 2-AG: neuron firing rate in response to stimulus in rat hippocampal neurons. At low firing rates, THC mimicked 2-AG and behaved like an agonist; at high firing rates, THC antagonized endogenous 2-AG signaling.

AEA is a partial agonist like THC, with an efficacy somewhat greater than THC in mouse brain [308] and transfected human CB_1_ [296]. Consistent with partial agonism, exogenously-administered AEA caused little tolerance in rodents [309], [310]. Agonist trafficking adds further complexity—THC and AEA preferentially activate different G-protein subtypes [311]. At transfected human CB_1_, AEA acted as a full agonist via Gαi subunits, and a partial agonist via Gαo subunits, with agonist efficacy much greater than THC at Gαi, and slightly greater than THC at Gαo [312].

AEA and THC can antagonize each other; this in part is due to cross-tolerance [313], [314]. Falenski *et al.* [287] demonstrated that subchronic administration of THC in FAAH^−/−^ knockout mice caused greater tolerance to THC than did subchronic administration of THC in wildtype mice. Thus elevated levels of AEA in FAAH^−/−^ knockouts produced additive effects with THC. Vann *et al.* [315] trained rats to discriminate THC; trained rats injected with PMSF, which inhibits FAAH, showed 2.7-fold greater discrimination than rats injected with vehicle. In other words, inhibiting AEA degradation led to an increase in the potency of THC. Further, THC was more potent at producing antinociception, decreasing spontaneous activity, and increasing ring immobility when co-administered with PMSF as compared to vehicle.

In summary, the effects of THC upon the eCB system oscillate between potentiation and suppression, depending on acute versus chronic dosage. The dividing line between “acute” and “chronic” is a gray zone, and likely differs amongst individuals. Suplita *et al.* [316] summarized the situation: they studied “stress antinociception,” where rodents become less responsive to painful stimuli following exposure to an environmental stressor. Stress antinociception is mediated, in part, by the coordinated release of 2-AG and AEA. Acute administration of THC potentiated eCB-mediated stress antinociception. The converse was also true: animals exposed acutely to foot shock, which elicits eCB-mediated stress antinociception, became sensitized to the effects of THC. Chronic administration of THC predictably dampened stress antinociception. The converse was *not* true: chronic exposure to foot shock (3 min/day for 15 days) failed to dampen antinociception induced by either WIN-55,212-2 or by further footshocks.

The potential synergy between THC and the eCB system is analogous to the potential synergy between AEA and 2-AG: Rodent studies that combined FAAH and MAGL inhibitors indicated that AEA and 2-AG may activate CB_1_ receptors in different parts of the central nervous system. Each causes unique behavioral effects, and when both are enhanced, new effects emerge. Long and colleagues [317] showed that AEA and 2-AG independently dampen pain sensation, but together their effects are dramatically enhanced.

Cannabis is more than THC [318], [319]. Adding CBD to THC in mice enhanced CB_1_ expression in hippocampus and hypothalamus [320]. CBD increased hippocampal cell survival and neurogenesis, whereas THC had the opposite effect; the CBD response was absent in CB_1_^−/−^ knockout mice [321]. CBD inhibited the cellular uptake of AEA and its breakdown by FAAH [322], [323]. A separate systematic review regarding the effects of CBD on THC is currently underway (McPartland, unpublished). Several other non-THC cannabinoids interact with enzymes of the eCB system. For example, cannabidivarin and cannabidiolic acid are moderately potent inhibitors of DAGLα, and cannabigerol and cannabichromene are relatively potent inhibitors of anandamide cellular uptake [323]. Interestingly, cannabis extracts (“botanical drug substances,” BDS) enriched in cannabinoids, such as THC-acid BDS and CBD-BDS, were more potent than the corresponding pure compounds at inhibiting MAGL and AEA cellular uptake [323].

### Conclusions

Many randomized controlled trials identified in this systematic review have been conducted on lifestyle modifications (*e.g.*, exercise, maintenance of ideal body weight) and CAM interventions (*e.g.*, dietary supplements, stress modification, acupuncture, massage and manipulation). In our opinion these are sensible methods of enhancing the eCB system.

Preclinical studies identified useful prescription drugs, such as SSRIs, anxiolytics, antipsychotics, and anticonvulsants. However, these drugs are generally administered in a chronic fashion, and this comes with a caveat: generating chronic elevations in AEA and 2-AG may be counterproductive. Faced with constant activation by agonists, CB_1_ and CB_2_ desensitize and downregulate. A desensitized receptor drives less receptor-mediated signal transduction, and develops cross-tolerance to all agonists—eCBs and phytocannabinoids alike. A downregulated receptor is not functional—either it does not bind ligand or has internalized away from the cell membrane.

The difference between acute and chronic augmentation has been demonstrated in rodent studies: acute blockade of MAGL with JZL184 elevated 2-AG levels and provided analgesia [324]. In the face of chronic blockade with JZL184 this analgesia was lost, because sustained elevation of 2-AG caused CB_1_ desensitization. This led to a loss in eCB-dependent synaptic plasticity, cross-tolerance to other cannabinoids, and physical dependence.

Other drugs identified in preclinical studies have side effect profiles too severe to warrant their use for upregulating the eCB system (*e.g.*, corticosteroids, opioids, nicotine). Preclinical studies suggest a number of over-the-counter medications, such as analgesics, seem to be acting through eCB-mediated mechanisms. Clinical trials are warranted, although over-the-counter medications lack patent protection, so expensive clinical trials seem unlikely.

## Supporting Information

Checklist S1 **Online supporting material.** Preferred Reporting Items for Systematic Reviews and Meta-Analyses (PRISMA) checklist.
(DOC)
